# Case Report: Immune checkpoint inhibitor–induced multiorgan vasculitis successfully treated with rituximab

**DOI:** 10.3389/fneph.2023.1168614

**Published:** 2023-07-20

**Authors:** Sehrish Qureshi, Naszrin Arani, Vishnu Parvathareddy, Amanda Tchakarov, Maen Abdelrahim, Maria Suarez-Almazor, Jianjun Zhang, Don Lynn Gibbons, John Heymach, Mehmet Altan, Ala Abudayyeh

**Affiliations:** ^1^ Department of General Internal Medicine, Division of Internal Medicine, The University of Texas MD Anderson Cancer Center, Houston, TX, United States; ^2^ Section of Nephrology, Division of Internal Medicine, The University of Texas MD Anderson Cancer Center, Houston, TX, United States; ^3^ Department of Pathology and Laboratory Medicine, University of Texas Health Science Center McGovern Medical School, Houston, TX, United States; ^4^ Department of Medical Oncology, Institute of Academic Medicine and Weill Cornell Medical College, Houston Methodist Cancer Center, Houston, TX, United States; ^5^ Department of Health Services Research and Section of Rheumatology and Clinical Immunology, The University of Texas MD Anderson Cancer Center, Houston, TX, United States; ^6^ Department of Thoracic/Head & Neck Medical Oncology, The University of Texas MD Anderson Cancer Center, Houston, TX, United States

**Keywords:** immune check inhibitor, autoimmune induction, vasculitis, acute renal failure, rituximab

## Abstract

Immune checkpoint inhibitors (ICIs) have revolutionized the treatment of cancer. ICIs have a unique side effect profile, generally caused by inflammatory tissue damage, with clinical features similar to autoimmune conditions. Acute kidney injury from ICIs has been well studied; incidence ranges from 1% to 5%, with higher incidence when combination ICI therapies are used. Although the overall reported incidence of ICI-associated glomerulonephritis is less than 1%, vasculitis is the most commonly reported ICI-related glomerulonephritis. Other biopsy findings include thrombotic microangiopathy, focal segmental glomerulosclerosis, minimal change disease, and IgA nephropathy with secondary amyloidosis. We report a case in which a woman previously treated with the PD-L1 inhibitor durvalumab for locally advanced non-small cell lung cancer with pre-existing antineutrophil cytoplasmic (anti-PR3) antibody who later developed multi-organ vasculitis after ICI exposure, which was successfully treated with rituximab, with continued cancer remission for 3 years.

## Background

Immune checkpoint inhibitor (ICI) therapy targeting inhibitory proteins within the immune system, such as programmed cell death protein 1 and its ligand (PD-[L]1) and cytotoxic T-lymphocyte–associated protein 4 (CTLA-4), has resulted in significant improvements in disease outcomes for various cancers. A challenge of ICI therapy is the uninhibited and dysregulated immune response of various organs of the body, also known as immune-related adverse events (irAEs). irAEs have been associated with therapy-related mortality, overall mortality, and treatment interruptions or discontinuation leading to tumor progression. Therefore, a more targeted approach to the treatment of irAEs, in addition to steroids, is needed to prevent ICI discontinuation and improve outcomes.

The incidence of renal irAEs is reported to be only 1–5%; however, any renal compromise can reduce overall survival of patients regardless of disease severity and may reduce eligibility for further clinical trials (1). Therefore, close monitoring of kidney function is needed to preserve and optimize it during ICI therapy. Renal irAEs have varied presentations, including electrolyte disturbances, acute tubulointerstitial nephritis, acute tubular necrosis, vasculitis, glomerulonephritis, and, rarely, sarcoidosis. Although glomerulonephritis after ICI exposure is rare, it can complicate cancer treatment and therefore affect survival. In a recent meta-analysis of *de novo* glomerular diseases after ICI exposure, pauci-immune glomerulonephritis and renal vasculitis accounted for 27% of glomerular disease cases, followed by minimal change disease (20%) and C3 glomerulonephritis (11%), and 41% of the patients had concomitant acute tubulointerstitial nephritis (2). The median time to glomerular disease diagnosis after starting ICI therapy was 93 days (interquartile range 44-212 days); 98% of patients received corticosteroids, and 31% had complete recovery and 42% had partial recovery from acute kidney injury. Approximately 19% of the patients underwent dialysis, and of these, approximately one-third died.

The prevalence of autoantibody development after ICI exposure has been well established, although this occurs more commonly with endocrine, skin, and muscle irAEs than with renal irAEs (3). Patients with autoimmune diseases and cancer have been treated with ICIs with extreme caution for fear of reactivation or exacerbation of the autoimmune disease, but in a recent multicenter international study of 55 melanoma patients with autoimmune disease treated with combination ICI therapy, only 33% had a flare of their autoimmune disease, and outcomes did not differ between those who were receiving immunosuppressive therapy and those who were not (4). In the past few years, there has been a growing interest in identifying pre-existing antibodies in cancer patients treated with ICIs to predict the development of irAEs or survival (5, 6). However, results have been inconsistent; other studies have indicated that pre-existing autoantibodies were not associated with an increased risk of irAEs and did not affect tumor response to ICIs (7, 8). However, the autoantibodies evaluated in several of these studies did not include antineutrophil cytoplasmic antibodies (ANCA), either before or after ICI exposure.

Kidney biopsies are needed both to diagnose glomerulonephritis and to tailor treatment, and the use of rituximab has been effective in attaining remission (9, 10). We report a case in which a woman previously treated with the PD-L1 inhibitor durvalumab for locally advanced non-small cell lung cancer developed multi-organ vasculitis and was positive for ANCA. Her vasculitis was successfully treated with rituximab, with continued cancer remission for 3 years, suggesting that this approach may be useful in cancer patients with ICI-induced kidney injury and develop vasculitis.

## Case presentation

The patient was a 55-year-old white woman with a past medical history of intermittent thyroiditis, which had led to total thyroidectomy, and recurrent stage IIIA T2aN2M0 adenocarcinoma of the right lower lung. Upon initial diagnosis of lung cancer, she had undergone video-assisted thoracoscopic right lobectomy in October 2010, followed by 4 cycles of chemotherapy with cisplatin and vinorelbine 4, which was completed in May 2011, as well as adjuvant radiotherapy consisting of 50 Gy in 25 fractions for N2 disease, which was completed in August 2011. Restaging positron emission tomography/computed tomography (PET/CT) in October 2019 had shown FDG-avid soft tissue involvement in the right hilum and biopsy, confirming recurrent adenocarcinoma of the lung. The patient had received definitive chemoradiation, initiated in November 2019. She had completed chemoradiation in December and started consolidative anti-PD-1 therapy with durvalumab, as per standard of care.

**Figure 1:**
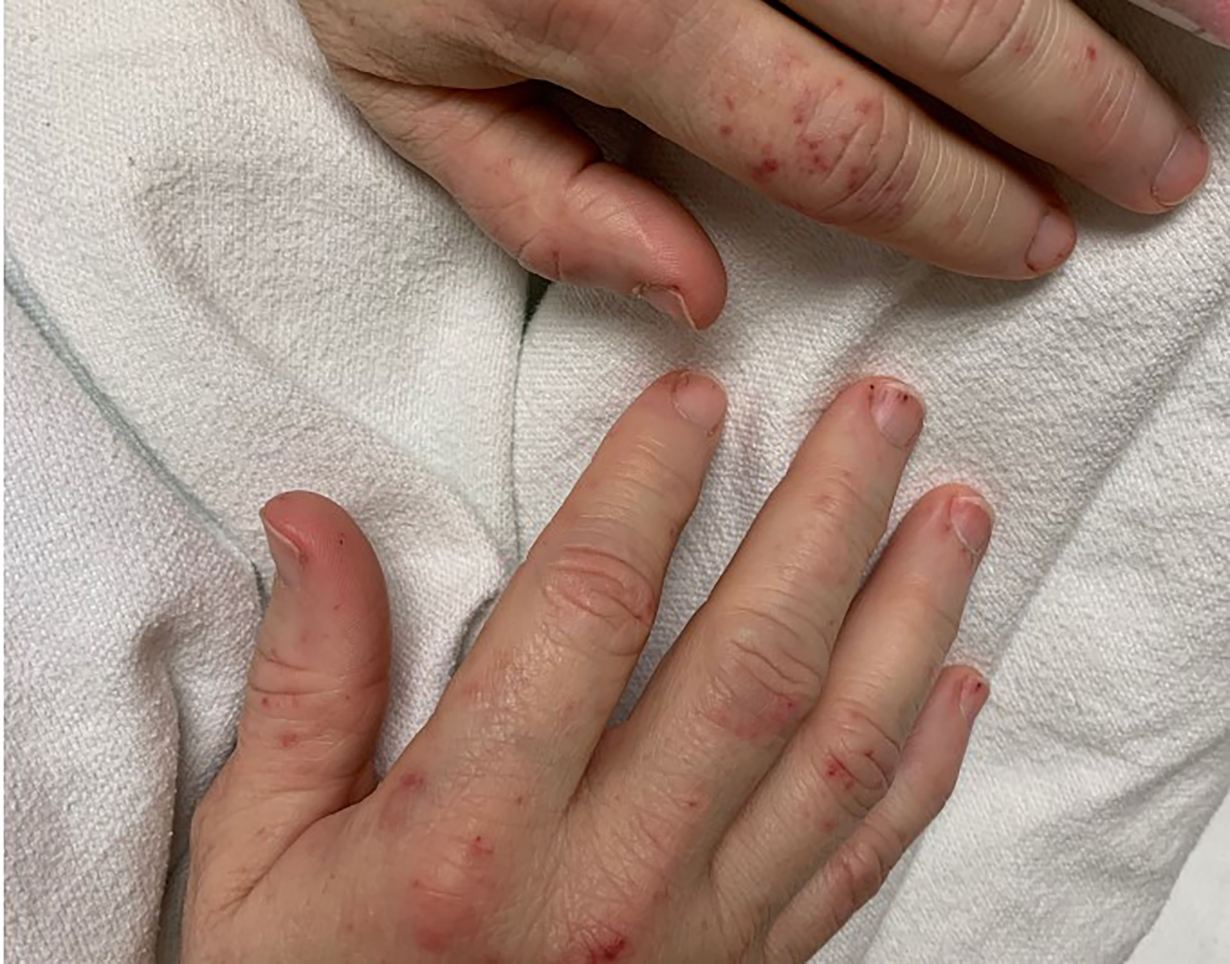
Splinter hemorrhages on bilateral hands.

Within 1 month of beginning ICI therapy, the patient developed oral blisters and high fevers, and she subsequently had hemoptysis, a productive cough, and acute kidney injury with hematuria and proteinuria of 2 g. Her serum creatinine was 0.56 mg/dL. Her physical examination was significant for tachycardia, vesicular lip lesions, minimal wheezing in the right upper lung field, and splinter hemorrhages and purpura of the fingers (**Figure 1**). Laboratory workup revealed positivity for herpes simplex virus-1 DNA and pneumonia sputum cultures positive for methicillin-sensitive *Staphylococcus aureus*. Clinical suspicion for endocarditis in the physical examination resulted in a transesophageal echocardiogram, which showed a small (<1-mm) echogenic structure attached to the atrial surface of the mitral valve. All blood cultures were negative. The patient was diagnosed with culture-negative infectious endocarditis and treated with cefazolin at a dose of 1 g intravenously every 12 hours (reflecting renal adjustment for kidney injury), which she continued for 6 weeks. Follow-up PET/CT indicated resolved uptake in the heart.

**Figure 2:**
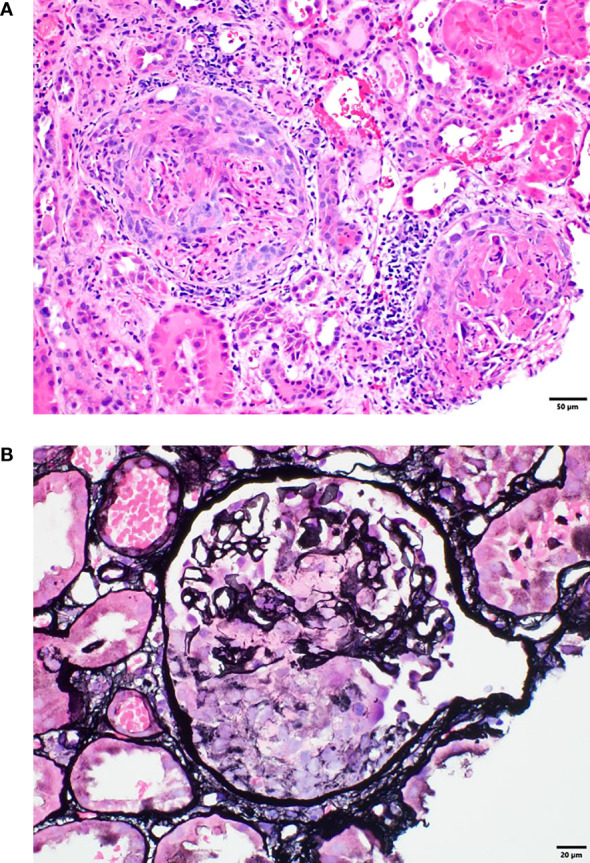
**(A)** Renal biopsy tissue with hematoxylin and eosin staining showing crescentic and necrotizing glomeruli. **(B)** Renal biopsy tissue with Jones silver staining showing a glomerulus with segmental necrosis and glomerular basement membrane disruption.

**Figure 3:**
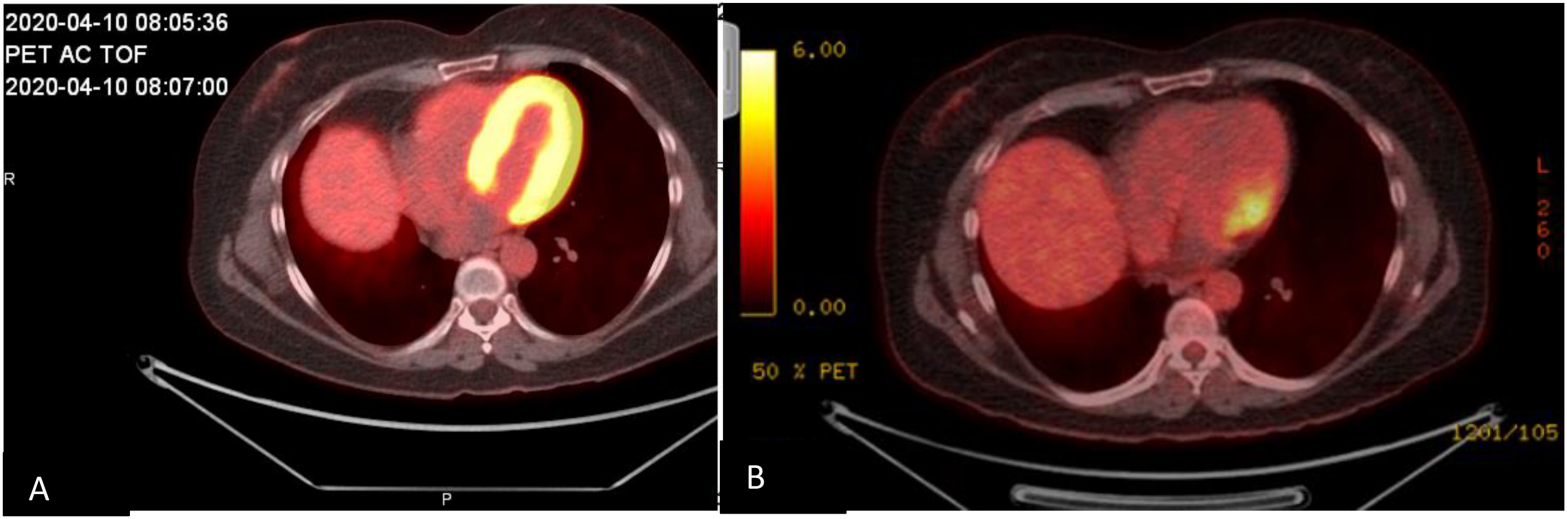
Cardiac positron emission tomography/computed tomography images obtained **(A)** during initial hospitalization, showing FDG activity surrounding the myocardium, and **(B)** 4 months after hospitalization, showing improvement of FDG activity surrounding the myocardium.

Because the patient had proteinuria and hematuria, she also underwent a kidney biopsy, which revealed acute pauci-immune segmental necrotizing (50%) and crescentic (36%) glomerulonephritis and acute tubular epithelial injury consistent with acute tubular necrosis. These findings were consistent with anti-PR3 ANCA–associated glomerulonephritis and/or systemic vasculitis. Examination of approximately 50 glomeruli in paraffin sections revealed 25 (50%) with segmental necrosis/endocapillary hypercellularity and 18 (36%) with necrosis with full crescents (**Figure 2**). The patient was aggressively treated with pulsed methylprednisolone at a dose of 500 mg for 3 days and plasmapheresis for 4 days. Durvalumab was permanently discontinued. She was treated with one dose (1 g) of rituximab, followed by a second dose 2 weeks after the first dose (**Figure 3**). Her baseline serum creatinine level of 0.56 mg/dL peaked at 3.22 mg/dL and improved to 1.0 mg/dL after treatment with rituximab.

**Figure 4:**
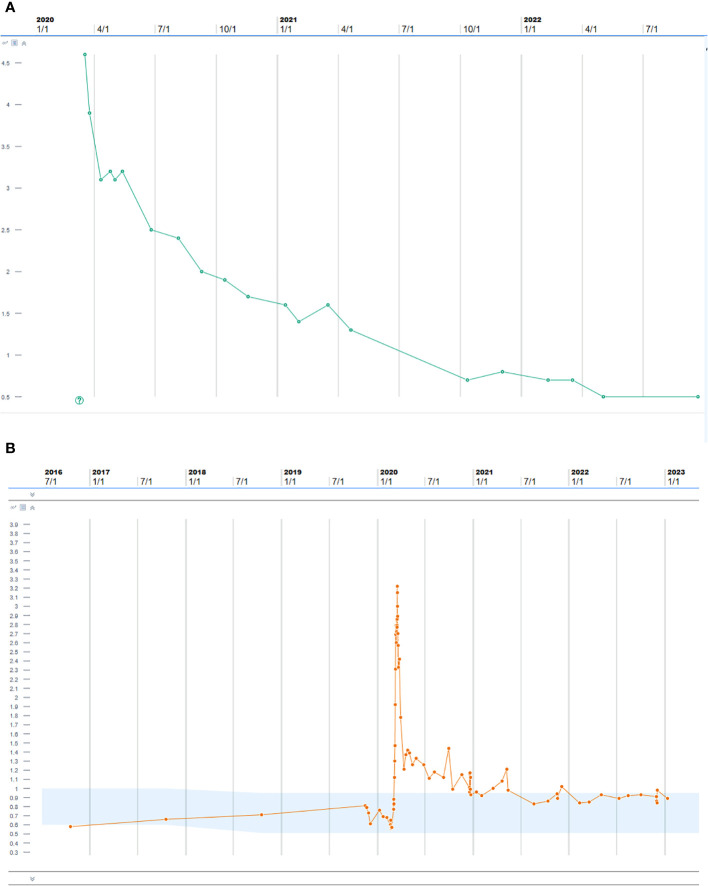
**(A)** Proteinase 3 antibody after treatment with pulse steroids, plasmapheresis, and rituximab. **(B)** Creatinine trend after treatment with pulse steroids, plasmapheresis, and rituximab.

The patient’s renal disease subsequently went into almost complete remission; most recently, in December 2022, her creatinine was 0.98 mg/dL with bland urine negative for ANCA (**Figure 4**). Her skin changes, lung wheezing, and suspected endocarditis had also resolved, and the cancer remained in remission at 2 years after discontinuing ICIs.

## Discussion

Over the past few years, a growing number of patients with relapsed/refractory cancer have qualified for therapy with ICIs, with around 45% of individuals now eligible for therapy (1, 2, 6). Thus, as ICIs are being used more frequently, irAEs associated with them are being reported at increasing rates, allowing us to learn more about the pathogenic mechanisms behind ICI-associated nephrotoxicity (2, 6). Although the mechanism by which ICIs cause acute kidney injury is not clearly understood, it has been theorized that ICIs may provoke T-cell infiltration within the renal tubulointerstitium or possibly cause memory T-cell activation via loss of immune tolerance, especially in patients with prior hapten exposure (i.e., proton pump inhibitors), thus causing unchecked activation of the immune system with subsequent kidney injury (11).

Similar to a recent case series reporting vasculitis after ICI exposure, our use of kidney biopsy in the current case is a departure from some of the current guidelines for management of renal irAEs that suggest forgoing kidney biopsy and starting corticosteroid-based therapy (10, 12).

Although development of glomerulonephritis can lead to interrupting or discontinuing use of ICIs, several case reports have indicated that treatment with rituximab allowed patients to continue receiving ICIs and achieve both renal and disease remission (2, 13).

Another possible mechanism inducing autoimmune disease in the kidney is ICI-induced regulatory T-cell suppression, which would thereby increase the risk for antibody-mediated autoimmune diseases and upregulation of interferon-alpha and interleukin-12. Upregulation of these two cytokines has been associated with increased B-lymphocyte interaction with expanded T-lymphocyte populations and development of ANCA vasculitis (14–18). Additionally, ICIs have been reported to upregulate CXCL9 and CXCL10, which facilitate T-cell recruitment and have been associated with tissue injury in patients with IgA vasculitis (19, 20).

In our experience, we recommend treatment with rituximab over other cytotoxic therapies for patients with glomerulonephritis and (non-crescentic) vasculitis. Rituximab, a monoclonal anti-CD20 antibody, disrupts pathogenic B-lymphocyte interaction with cytotoxic T lymphocytes, reduces chemokine production, and limits endothelial injury, and so far, rituximab has not been shown to inhibit the anti-neoplastic effects of ICI therapy (21, 22). In a small cohort of five patients diagnosed with ICI-induced renal vasculitis, treatment with rituximab resulted in partial to complete renal recovery and no renal relapses (10). We also recommend to avoid maintenance rituximab once remission of vasculitis is achieved since B cells are classically known to further the activation and proliferation of the T-cells through antigen presentation (23).

In the prospective multicenter PEXIVAS trial, plasmapheresis was not superior to other therapies in the treatment of severe ANCA-associated vasculitis accompanied by alveolar hemorrhage and/or kidney disease with an estimated glomerular filtration rate of 50 mL/minute per 1.73 m^2^. However, that trial did not evaluate the role of kidney histologic findings in predicting kidney function and response to plasma exchange (24). A recent paper by Nezam et al. evaluated pathologic findings and renal response to plasma exchange and noted that although renal outcomes did not differ between the 188 patients with ANCA-associated vasculitis and acute kidney injury treated with plasma exchange and the 237 not treated with plasma exchange, pathologic findings suggested that 42% of patients from this cohort could have benefitted from plasma exchange with a clinically relevant reduction of mortality and kidney injury. Factors indicating that plasma exchange could benefit patients included microscopic polyangiitis, MPO-ANCA positivity, high serum creatinine, crescentic and sclerotic classes, and high Brix score (25); these factors are consistent with our case. This indicates that patients receiving ICIs who present with profound autoimmune symptoms would benefit from plasmapheresis to remove the autoimmune antibodies (26).

Our patient had valvular involvement with negative blood cultures, which could have been mistaken for endocarditis if not for the kidney biopsy confirming ANCA-associated vasculitis. This was further confirmed by follow-up PET/CT showing resolution of uptake after several months of treatment with rituximab. In a recent literature review of pediatric patients with cardiac ANCA-associated vasculitis, three of the five patients had valvular involvement (27).

When rituximab is used in conjunction with corticosteroids, renal recovery rates are higher than those reported with corticosteroid monotherapy, and corticosteroid therapy duration can be shortened (2, 5). Our patient had grade 3 immune-mediated glomerulonephritis and biopsy results showing pauci-immune ANCA-positive disease, for which she received three pulses of 1,000 mg of intravenous methylprednisolone per day followed by prednisone at a dose of 1 mg/kg per day. The patient also was treated with plasmapheresis for aggressive management, followed by two doses of rituximab administered 2 weeks apart. In addition, her baseline serum samples prior to ICI exposure indicated that she was positive for ANCA, which was probably a paraneoplastic induction with the lung infiltration at baseline, later unleashing the underlying autoimmune disease after ICI exposure. No current data are available on whether ANCA positivity prior to ICI exposure is associated with induction of vasculitis, and therefore, screening prior to ICI use for autoantibodies specifically for ANCA is worthy of further study. Our patient has been followed closely every 3 months, and serum ANCA has remained negative.

In conclusion, ICIs have revolutionized the management of refractory/relapsing cancers. By activating the immune system using monoclonal anti-CTLA-4, PD-1, and PD-L1 antibodies, ICIs can yield durable antitumor responses and augment survival. However, ICI-induced vasculitis is a known adverse effect of ICIs that clinicians must consider when managing an acute kidney injury in a patient who has received one of these agents. Whether ICI-induced vasculitis is limited to one organ or systemic, it is associated with considerable morbidity (10). Therefore, it is critical that clinicians remain vigilant for ICI-induced vasculitis by monitoring serum creatinine levels after initiation of therapy, during treatment, and up to 12 months thereafter (9). Treating ICI-induced immune-mediated nephritis with corticosteroids, plasmapheresis, and rituximab can potentially lead to renal recovery, as it did in our patient.

## Data availability statement

The original contributions presented in the study are included in the article/supplementary material. Further inquiries can be directed to the corresponding author.

## Ethics statement

The studies involving human participants were reviewed and approved by The Institutional Review Board at The University of Texas MD Anderson Cancer Center. The patients/participants provided their written informed consent to participate in this study. Written informed consent was obtained from the participant/patient(s) for the publication of this case report.

## Author contributions

AA: concept/design, interpretation, drafting article, critical revision of article, approval of article, data collection, and secured funding. SQ, NA, and VP: drafting article, interpretation, critical revision of article, approval of article, collection of data. AT, MaA, MS-A: interpretation, drafting article, critical revision of article, approval of article, and final approval. JZ, DG, JH, MeA data collection, critical revision of article, and approval of article, funding support.
